# Oligo targeting for profiling drug resistance mutations in the parasitic trypanosomatids

**DOI:** 10.1093/nar/gkac319

**Published:** 2022-05-07

**Authors:** Simone Altmann, Eva Rico, Sandra Carvalho, Melanie Ridgway, Anna Trenaman, Hannah Donnelly, Michele Tinti, Susan Wyllie, David Horn

**Affiliations:** The Wellcome Trust Centre for Anti-Infectives Research, School of Life Sciences, University of Dundee, Dow Street, Dundee DD1 5EH, UK; The Wellcome Trust Centre for Anti-Infectives Research, School of Life Sciences, University of Dundee, Dow Street, Dundee DD1 5EH, UK; The Wellcome Trust Centre for Anti-Infectives Research, School of Life Sciences, University of Dundee, Dow Street, Dundee DD1 5EH, UK; The Wellcome Trust Centre for Anti-Infectives Research, School of Life Sciences, University of Dundee, Dow Street, Dundee DD1 5EH, UK; The Wellcome Trust Centre for Anti-Infectives Research, School of Life Sciences, University of Dundee, Dow Street, Dundee DD1 5EH, UK; The Wellcome Trust Centre for Anti-Infectives Research, School of Life Sciences, University of Dundee, Dow Street, Dundee DD1 5EH, UK; The Wellcome Trust Centre for Anti-Infectives Research, School of Life Sciences, University of Dundee, Dow Street, Dundee DD1 5EH, UK; The Wellcome Trust Centre for Anti-Infectives Research, School of Life Sciences, University of Dundee, Dow Street, Dundee DD1 5EH, UK; The Wellcome Trust Centre for Anti-Infectives Research, School of Life Sciences, University of Dundee, Dow Street, Dundee DD1 5EH, UK

## Abstract

Trypanosomatids cause the neglected tropical diseases, sleeping sickness, Chagas disease and the leishmaniases. Studies on these lethal parasites would be further facilitated by new and improved genetic technologies. Scalable precision editing methods, for example, could be used to improve our understanding of potential mutations associated with drug resistance, a current priority given that several new anti-trypanosomal drugs, with known targets, are currently in clinical development. We report the development of a simple oligo targeting method for rapid and precise editing of priority drug targets in otherwise wild type trypanosomatids. In *Trypanosoma brucei*, approx. 50-b single-stranded oligodeoxynucleotides were optimal, multiple base edits could be incorporated, and editing efficiency was substantially increased when mismatch repair was suppressed. Resistance-associated edits were introduced in *T. brucei* cyclin dependent kinase 12 (CRK12, L^482^F) or cleavage and polyadenylation specificity factor 3 (N^232^H), in the *Trypanosoma cruzi* proteasome β5 subunit (G^208^S), or in *Leishmania donovani* CRK12 (G^572^D). We further implemented oligo targeting for site saturation mutagenesis, targeting codon G^492^ in *T. brucei* CRK12. This approach, combined with amplicon sequencing for codon variant scoring, revealed fourteen resistance conferring G^492^ edits encoding six distinct amino acids. The outputs confirm on-target drug activity, reveal a variety of resistance-associated mutations, and facilitate rapid assessment of potential impacts on drug efficacy.

## INTRODUCTION

The parasitic trypanosomatids cause a range of lethal and neglected tropical diseases. These protozoa include *Trypanosoma brucei* spp*, Trypanosoma cruzi* and *Leishmania* spp, which cause sleeping sickness, Chagas disease and leishmaniasis (both visceral and cutaneous) in humans, respectively, while *T. brucei brucei* causes nagana in livestock (1). All three human diseases have been targeted for elimination as part of the World Health Organisation's road map for neglected tropical diseases 2021–2030 (WHO/UCN/NTD/2020.01).

Encouragingly, several new drugs with known mechanisms of action and known primary targets are in clinical development, promising to deliver new, safe, affordable, efficacious and often, oral therapies. These new priority targets include cyclin dependent kinase 12 (CRK12) (2,3), cleavage and polyadenylation specificity factor 3 (CPSF3) (4) and the proteasome (5,6). Since drug resistance can undermine both the efficacy of new therapies and substantial investments in drug discovery and clinical development, there is an urgent need for further insights into resistance-associated mutations and structure–activity relationships.

A conventional approach often used to identify the target of an anti-parasitic compound involves exposing populations of cells to drug pressure, for up to 6 months, followed by sequencing the genomes of drug-resistant parasites. Indeed, this approach revealed mutations in the genes encoding *Leishmania donovani* CRK12 (2), *T. cruzi* proteasome β5 (5) and *L. donovani* proteasome β4 or β5 subunits (6), contributing towards identifying these proteins as drug targets. Notably though, these approaches typically reveal single point mutations, which can sample only a limited subset of alternative amino acids, contingent upon mutational space (7).

CRISPR Cas9 RNA-guided nucleases have been successfully employed for genome editing in trypanosomatids (8–13) and in other parasitic protozoa (14). Notwithstanding their broad utility, the associated DNA double-strand breaks are cytotoxic and can increase off-target mutation rates (15), by several hundred-fold in regions adjacent to a break (16). RNA-guided deaminases and base editors (17), and also prime editors (18), display much lower rates of off-target editing, but use of these editors has not yet been reported in trypanosomatids. In addition, a protospacer-adjacent motif (PAM) must typically be present close to a CRISPR Cas9 editing site. Oligo targeting is an alternative genome editing approach that has not previously been developed for parasitic protozoa.

Genome editing using oligo targeting in *Saccharomyces cerevisiae* involves incorporation of synthetic single-stranded DNA (ssDNA) oligodeoxynucleotides (ssODNs) on the lagging strand of the DNA replication fork, favoured by discontinuous replication on this strand (19). Consequently, the approach is not thought to create DNA breaks or nicks and does not require a proximal PAM. Previously deployed in bacteria, yeast, plant and mammalian cells, this approach facilitates targeted chromosome modification at single base-pair resolution. Indeed, the approach is scalable in *Escherichia coli* (20) and *S. cerevisiae* (19) and facilitates multiplex genome engineering in these cells. Given the simplicity and potential versatility of oligo targeting, we sought to develop this technology and to apply it to the parasitic trypanosomatids.

## MATERIALS AND METHODS

### Oligo targeting in *T. brucei*

Bloodstream form *T. brucei* Lister 427 wild type cells were grown in HMI-11 medium in a humidified incubator at 37°C with 5% CO_2_. Optimally, 2.5 × 10^7^*T. brucei* cells were mixed with 100 μl of Nucleofector buffer (Amaxa T -cell kit) and then mixed with the appropriate ssODN (Thermo Fisher Scientific, except for LNA-modified ssODNs; Integrated DNA Technologies), 40 μg in 10 μL of 10 mM Tris–Cl, pH 8.5. The mixture was placed in a 0.2 cm gap electrocuvette and submitted to a single pulse using a Nucleofector (Amaxa) set on the Z-001 programme. The mixture was then transferred from the cuvette to 20 ml of prewarmed HMI-11 medium and placed in the incubator. After 6 h, the appropriate drug selection was applied, in excess of the previously determined EC_50_ and sufficient to eliminate mock-transfected cells; 10 nM unless stated otherwise in the case of cpd 2 (3) and 1 μM in the case of acoziborole (4). The cells were then distributed in multi-well plates for sub-cloning or were expanded in bulk-culture for amplicon-seq profiling, with drug selection maintained in each case. Estimates of oligo targeting efficiency were derived by counting positive wells in minimally diluted plates or, when efficiency was high, in serially diluted plates, 5–6 days after plating.

### MSH2 knockdown by RNA interference

Initial analysis using *msh2* null strains (21) consistently yielded cells that tolerated drug selection. Since we were concerned that many mutations may have accumulated in these cells due to the prolonged mismatch repair defect, we adopted a conditional knockdown strategy. PCR primers, for amplification of the *T. brucei MSH2* (Tb927.10.11020) RNAi target fragment, were designed using RNAit (https://dag.compbio.dundee.ac.uk/RNAit/). Following PCR amplification, using *T. brucei* genomic DNA as template, the product was digested with BamHI and XmaI and ligated to similarly digested pRPa^iSL^ plasmid (22). In the second step, additional PCR product was digested with HindIII and ApaI and ligated to similarly digested pRPa^iSL^ plasmid from step 1. Correct assembly was confirmed by DNA sequencing. The *T. brucei MSH2* RNAi strain was constructed by digesting the final pRPa^MSH2-RNAi^ construct with AscI and electroporation into 2T1 *T. brucei* cells (23) as described above. After 6 h, phleomycin and hygromycin selection were applied, both at 2 μg/ml. The cells were then distributed in multi-well plates for sub-cloning. A puromycin-sensitive (2 μg/ml) sub-clone was selected for further analysis and subsequently maintained in phleomycin and hygromycin, both at 1 μg/ml. Knockdown by RNAi was induced by addition of tetracycline at 1 μg/ml to the growth medium. MSH2 knockdown was assessed using qRT-PCR. RNA was extracted using the RNeasy Mini Kit with an on-column DNase digest step (RNase-free DNase, Qiagen). RNA (1 μg) was reverse-transcribed to produce cDNA (M-MLV reverse transcriptase, Promega) and the equivalent of 25 ng of RNA was used in each qPCR reaction. The reactions were performed in technical triplicates on a QuantStudio3 system (Applied Biosystems) using the Luna® Universal qPCR Master Mix (NEB). TERT (Tb927.11.10190) and PFR (Tb927.8.5010) were used as reference genes (24) to calculate the 2^-deltaCt value for each sample.

### Oligo targeting in *T. cruzi*

Oligo targeting was carried out as above for *T. brucei*, but with the following modifications. Wild type *T. cruzi* [MHOM/BR/78/Silvio; clone X10/7A] epimastigotes were grown in RTH medium [RPMI 1640 medium supplemented with trypticase, haemin and HEPES] plus 10% heat-inactivated FBS in a humidified incubator at 28°C with 5% CO_2_. 2.5 × 10^7^*T. cruzi* cells were electroporated with the Nucleofector set on the X-014 programme. The mixture was then transferred from the cuvette to 10 mL of prewarmed medium and placed in the incubator. Cpd 7 (6) selection was applied at 350 nM after 24 h and thereafter. The cells were expanded in bulk-culture for 2–3 weeks, at which point no live cells were detectable in mock electroporated cultures (no oligo), and then distributed in multi-well plates for sub-cloning.

### Oligo targeting in *L. donovani*

Oligo targeting was carried out as above for *T. brucei*, but with the following modifications. Wild type *L. donovani* BOB strain [from MHOM/SD/62/1S-CL2D] promastigotes were grown in LdBOBpro (M199) medium in a humidified incubator at 28°C with 5% CO_2_. 2.5 × 10^7^*L. donovani* cells were electroporated with the Nucleofector set on the V-033 programme. The mixture was then transferred from the cuvette to 25 ml of prewarmed medium and placed in the incubator. Cpd 5 (2) selection was applied at 10 nM after 24 h, and cells were distributed in multi-well plates. Estimates of oligo targeting efficiency were derived by counting positive wells in minimally diluted plates, 14–16 days after plating. No drug-resistant cells were recovered from mock electroporation experiments (no oligo).

### Confirmation of edits using DNA-sequencing

*T. brucei* DNA was extracted using a DNeasy blood and tissue Kit (Qiagen). *Leishmania* and *T. cruzi* cells were harvested and resuspended in lysis buffer [10 mM Tris–HCl, pH 8, 100 mM NaCl, 25 mM EDTA, 0.5% (w/v) SDS, 0.1 mg/ml proteinase K]. After overnight incubation at 56°C, DNA was extracted using the phenol:chloroform:isoamyl alcohol method. PCR was used to amplify an appropriate segment from each edited gene, which was subjected to Sanger DNA sequencing. PCR primer pairs are detailed in Supplementary Table S1. A minimum of two independent clones were sequenced in each case and all sequences revealed the expected edits.

### Amplicon sequencing and analysis

*T. brucei* DNA was extracted as above; after 5 days of drug selection or after 24 h following mock transfection. A 176-bp PCR amplicon spanning the oligo targeted region was PCR-amplified using KOD Hot Start DNA Polymerase (Merck) and further processed using a PCR Purification Kit (Qiagen). Amplicon sequencing was then carried out using DNBseq at BGI Genomics. Briefly, rolling-circle amplification was used to produce DNA nanoballs followed by sequencing by combinatorial probe-anchor synthesis in 100 b paired-end mode on the BGISEQ-500 platform (The Beijing Genome Institute). Sequencing data were filtered using SOAPnuke software (BGI) with the following parameters: -n 0.001 -l 10 -q 0.4 -A 0.25 -Q 2 -G –cutAdaptor –minLen 100, to obtain between 17 and 30 million 100 b paired-end reads per sample. Reads were analysed with FastQC (https://www.bioinformatics.babraham.ac.uk/projects/fastqc/) and filtered with fastp (https://github.com/OpenGene/fastp) using default options. The reference genome was assembled using v46 of the *T. brucei* reference genome, clone 427_2018, downloaded from TriTrypDB (25). Forward and reverse paired-end reads were aligned to the reference genome using Bowtie 2 (26), with the ‘very-sensitive-local’ pre-set alignment option. The alignments were converted to BAM format, reference sorted and indexed with SAMtools (27). The quality of alignments was evaluated with Qualimap 2 (28) using the bamqc and rnaseq options. The Qualimap2 output files, and the outputs of fastp, bowtie2, Picard Mark Duplicates (https://broadinstitute.github.io/picard/), SAMtools flagastat, SAMtools stats were aggregated with MultiQC (29) and inspected. Per-codon read counts were derived for ten codons at the 5′ and 3′ of the degenerate codon using a custom python script implemented in a jupyter notebook. Briefly, the script parses the aligned reads in the bam file to retrieve the codon counts. The genomic coordinates of the aligned reads are first used to extract the codons starts/ends and to reconstruct the codon sequence. Reads with a quality score <30 in the analysed region were discarded from the counts. The scripts to align the fastq files, qc control files and the scripts to extract the codon counts are available at https://github.com/mtinti/DH_Oligo-targeting and deposited in Zenodo (10.5281/zenodo.5139694).

### Dose-response assays

Half maximal effective drug concentrations (EC_50_) were measured by seeding mid-log cells in 96-well plates in a 2-fold dilution series of the appropriate drug; 10^3^ cells/ml for *T. brucei*, 5 × 10^5^ cells/ml for *T. cruzi* and 5 × 10^4^ cells/ml for *Leishmania*. Resazurin sodium salt (Sigma) in PBS was then added to each well; 500 μM after 72 h growth for *T. brucei*, 500 μM after 96 h growth for *T. cruzi* and 50 μM after 72 h growth for *Leishmania*. The plates were incubated in resazurin prior to analysis; 6 h for *T. brucei*, 24 h for *T. cruzi* and 2–3 h for *Leishmania*. Fluorescence was then measured using an Infinite 200 pro plate reader (Tecan) at an excitation wavelength of 540 nm and an emission wavelength of 590 nm. Data were analysed using Prism (GraphPad).

## RESULTS AND DISCUSSION

### Establishing and optimising oligo targeting in *T. brucei*

While assessing a CRISPR Cas9 RNA-guided nuclease system to drive ssODN-templated editing in *T. brucei*, we consistently observed the rapid emergence of edited populations in control experiments combining wild type trypanosomes with a repair template but lacking CRISPR Cas9 components. This suggested, although not reported previously, that ssODN-directed mutagenesis, or oligo targeting could be an effective method for genetically manipulating these parasites.

**Figure 1.**
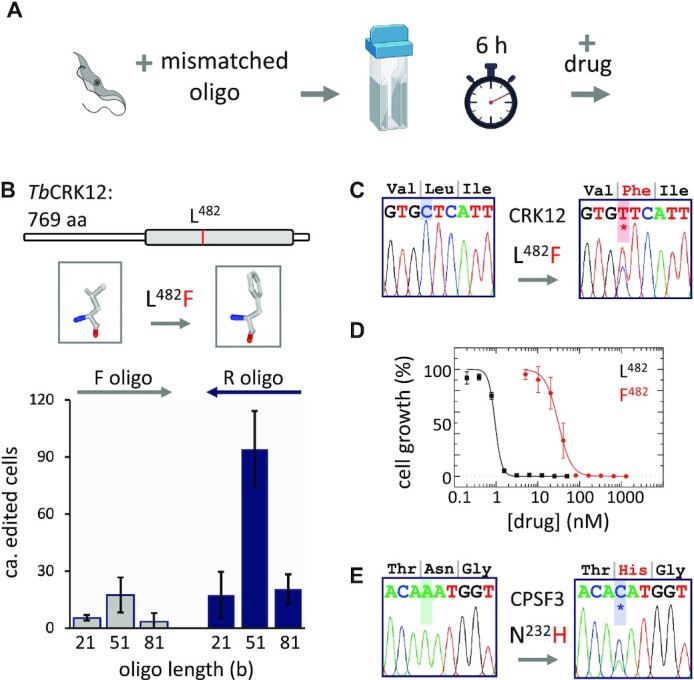
Establishing and optimising oligo targeting in *T. brucei*. (**A**) The schematic (created using BioRender.com) illustrates the oligo targeting protocol. (**B**) L^482^F, C-T editing in the kinase domain (grey bar) of *Tb*CRK12. The plot shows editing efficiency in *T. brucei* using ssODNs of different lengths in the forward (F) or reverse (R) orientation. A control lacking an ssODN failed to yield any drug-resistant clones. Compound 2 selection was applied at 10 nM; *n* = 2 for each assay; error bars, SD. (**C**) DNA sequencing confirmed the desired edit (also see Supplementary Figure S2A). (**D**) The representative dose-response curves shows that edited cells are drug-resistant; two independent clones were assessed; error bars, SD. (**E**) N^232^H, A–C editing of *Tb*CPSF3. DNA sequencing confirmed the desired edit (also see Supplementary Figure S2B).

The oligo targeting protocol (Figure 1A) that emerged employs wild type parasites mixed with a complementary but mismatched ssODN and subjected to a single pulse of electroporation. We first attempted to modify *T. brucei* CRK12 by introducing a L^482^F, CTC-TTC edit in the ATP binding site; this mutation preserves catalytic function and ATP-binding but confers resistance to compound 2 (3). We tested ssODNs of 21, 51 or 81 b in length and in either orientation, each with a centrally located mismatch. The drug was added six hours after electroporation and cells were distributed in multi-well plates, which were scored for clonal populations five to six days later. Selected clones were assessed for editing and drug resistance. This first set of six ssODNs tested yielded from ca. 3–94 drug-resistant clones. In terms of length, the 51 b ssODNs yielded the most clones (Figure 1B) and this was also the case compared to 41 or 61 b ssODNs (Supplementary Figure S1A), indicating a lower optimal ssODN length relative to *E. coli* and *S. cerevisiae*, where 90 b ssODNs are optimal (19,20). In terms of orientation, reverse ssODNs relative to the direction of transcription, and likely also DNA replication (30), yielded more clones (Figure 1B). This is consistent with insertion of complementary ssODNs on the lagging strand of the chromosomal DNA replication fork (19). DNA sequencing (Figure 1C) confirmed the desired heterozygous edit (trypanosomatids are diploid), which was associated with drug resistance (Figure 1D). We further validated the approach by introducing a N^232^H, AAT-CAT edit in *T. brucei* CPSF3 (Figure 1E), which creates a steric clash with acoziborole and confers resistance to this drug (4). No further base-changes were observed in the ssODN-complementary regions in either *Tb*CRK12 or *Tb*CPSF3 (Supplementary Figure S2A, B). These results indicate that oligo targeting is a precise gene-editing approach in *T. brucei* and that editing efficiency is both ssODN-length and ssODN-direction dependent.

### Multi-base editing and suppression by mismatch repair

**Figure 2.**
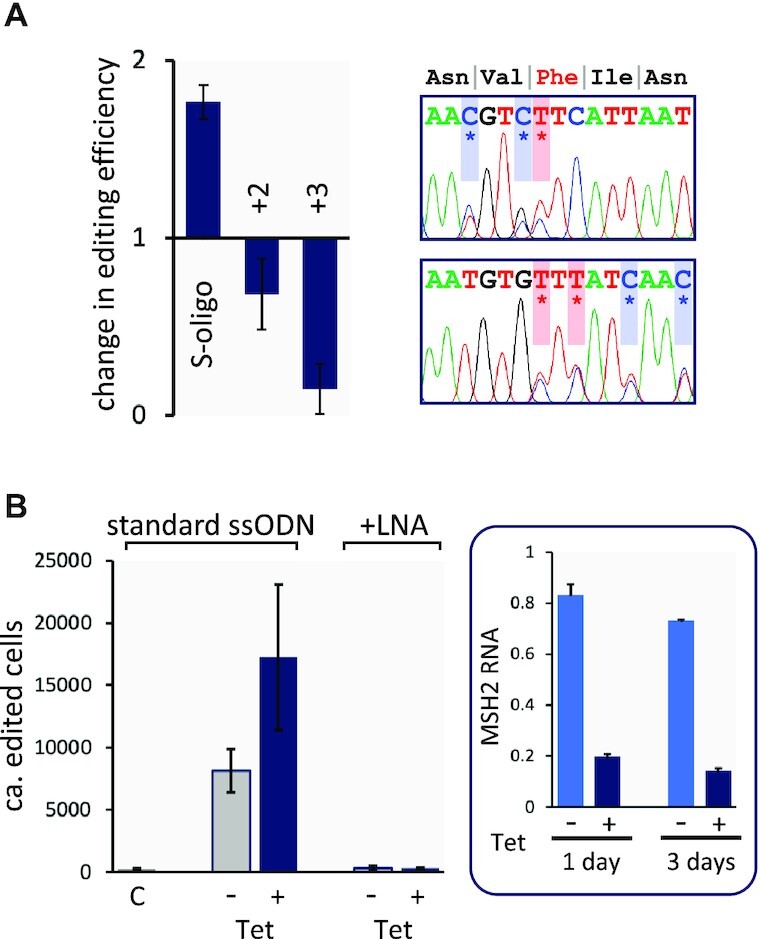
Multi-base editing and suppression by mismatch repair. (**A**) *Tb*CRK12 L^482^F editing efficiency using a 51-b reverse S-oligo containing three phosphorothioate internucleotide linkages at each end or using standard ssODNs with 2–3 additional changes. DNA sequencing confirmed the desired edits (also see Supplementary Figure S2A). (**B**) Assessment of the impact of mismatch repair. *Tb*CRK12 L^482^F editing efficiency following MSH2 knockdown using RNA interference; with either a standard 51-b reverse ssODN or with an equivalent ssODN containing a single locked nucleic acid (LNA) at the editing site. C, control; *T. brucei* cells lacking the RNAi construct. Another control following MSH2 knockdown but lacking the ssODN failed to yield any drug-resistant clones. The box on the right shows MSH2 knockdown relative to wild type cells as determined using qRT-PCR; 20–30% -Tet and >80% +Tet. Compound 2 selection was applied at 10 nM and *n* = 2 for each assay in A and B; error bars, SD.

We next asked whether the efficiency of oligo targeting could be improved in *T. brucei* and also whether multiple base changes could be incorporated in one step, again by introducing a CRK12–L^482^F edit. ssODNs with nuclease-resistant phosphorothioate internucleotide linkages (S-oligos) (31), also used for oligo targeting in *E. coli* (20), improved editing efficiency ∼2-fold (Figure 2A). A pair of ssODNs with two or three additional synonymous edits either 4 or 8 b apart, respectively, both yielded drug-resistant clones that incorporated all base-edits present in the ssODN; editing efficiency was reduced by 30 and 85%, respectively (Figure 2A). The protocol was next further optimised in terms of ssODN concentration and cell number (Supplementary Figure S1B). Given an electroporation survival rate of 44% (±5%, SEM) and an estimate of 342 edited clones per electroporation, we derive an optimal allele replacement frequency of 3 × 10^–5^ or 0.003% for these experiments. Finally, we asked whether terminal phosphate groups would impact oligo-targeting efficiency and found that these groups had only moderate impacts; a 5′-phosphate, which is required for ligation, reduced efficiency by ∼25%, whereas a 3′-phosphate, which can block extension by DNA polymerase, increased efficiency by ∼65% (Supplementary Figure S1C). These results suggested that neither direct ssODN ligation nor extension are required for oligo targeting. A 5′-phosphate may promote ssODN ligation, moderately limiting template molecule abundance and diffusion to target sites, while a 3′-phosphate may promote ssODN stability.

Editing by oligo targeting is suppressed by the mismatch repair (MMR) machinery in *E. coli* (32), *S. cerevisiae* (33), and in embryonic stem cells (34). Indeed, MMR targets the newly replicated DNA strand (33,35). Using *T. brucei* cells in which the MMR component MSH2 (21) was knocked down, we observed a 68-fold increase in ssODN-dependent generation of drug-resistant clones relative to control cells (Figure 2B). The output was also increased by 32-fold in cells grown under non-inducing conditions, however, which was likely due to 20–30% ‘leaky’ MSH2 knockdown (Figure 2B, inset). Thus, editing by oligo targeting is also suppressed by MMR in *T. brucei* and an allele replacement frequency of 1.6 × 10^–3^ or 0.16% can be achieved following MSH2 knockdown.

We also tested an ssODN containing a locked nucleic acid, which can preclude MMR protein binding in *E. coli* (36). The results indicated that oligo targeting efficiency is indeed unaffected by MMR when using this modified ssODN in *T. brucei* (Figure 2B). However, this ssODN yielded relatively low efficiency editing, both before and after MSH2 knockdown, suggesting that the locked nucleic acid also interferes with incorporation at the replication fork in *T. brucei*.

We concluded that oligo targeting in *T. brucei* was optimal when combining 25 million cells with 40 μg of a 50–55-b reverse or ‘antisense’ ssODN, stabilized with phosphorothioate bonds, and with 1–3 centrally located and closely clustered base-edits. Editing efficiency can be further increased by perturbing the native mismatch repair machinery.

### Establishing oligo targeting in *T. cruzi* and *Leishmania*

**Figure 3.**
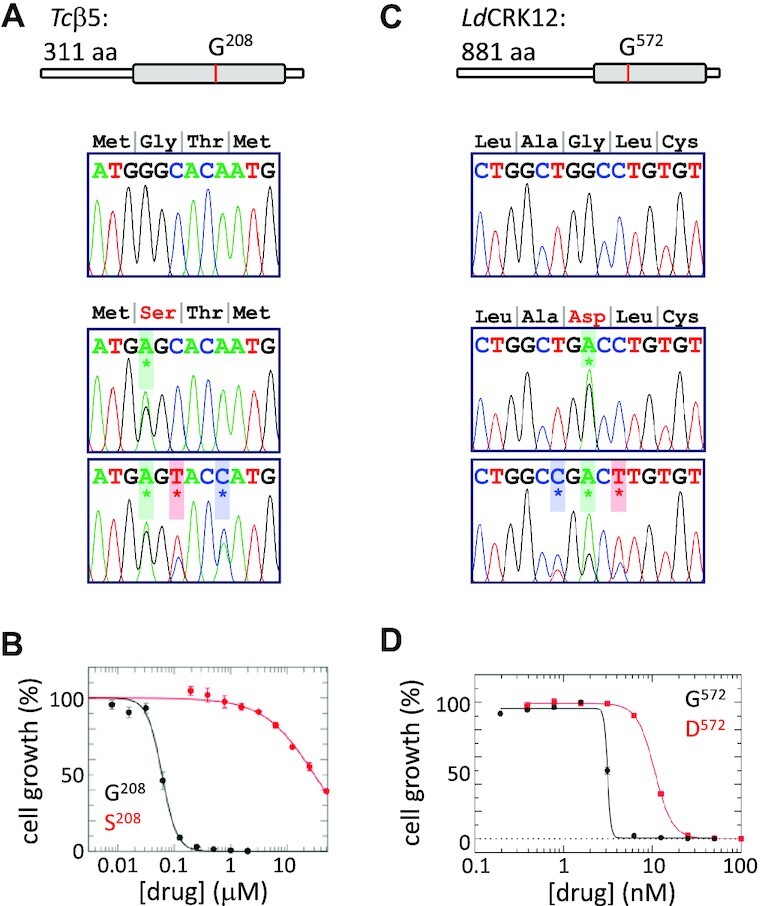
Establishing oligo targeting in *T. cruzi* and *Leishmania*. (**A**) G^208^S editing in the core domain (grey bar) of the *T. cruzi* proteasome β5 subunit, using either a 51-b reverse ssODN with a single G-A edit or a 56-b reverse ssODN with two additional edits. Compound 7 selection was applied at 350 nM. DNA sequencing confirmed the desired edits, *n* = 2 (also see Supplementary Figure S2C). A control lacking an ssODN failed to yield any drug-resistant clones. (**B**) The representative dose-response curves show that *T. cruzi* with the proteasome β5 G^208^S edit are drug-resistant; data are shown for one representative clone from three independent clones analysed; error bars, SD from two technical replicates. (**C**) As in A but showing G^572^D editing in the *L. donovani* CRK12 kinase domain (grey bar). In this case, a 55-b reverse ssODN was used to incorporate two additional synonymous edits. Compound 5 selection was applied at 10 nM (also see Supplementary Figure S2D). (**D**) As in B but for *L. donovani* CRK12 G^572^D editing; data are shown for one representative clone from two independent clones analysed; error bars, SD from two technical replicates. Similar results were obtained using S-oligos with a single mismatch.

Having established and optimised oligo targeting in *T. brucei*, we next sought to apply the technology to other trypanosomatid parasites. In *T. cruzi*, we targeted the proteasome β5 subunit and introduced a G^208^S, GGC-AGC edit, not reported previously but equivalent to the G^197^S mutation in the ligand binding pocket reported to produce a steric clash with, and resistance to, compound 7 in *L. donovani* (6). DNA sequencing confirmed the desired edit (Figure 3A), which was associated with drug resistance (Figure 3B), as well as the ability to introduce further adjacent edits (Figure 3A, Supplementary Figure S2C).

In *L. donovani*, we targeted CRK12 and introduced a G^572^D, GGC-GAC edit, which conferred resistance to compound 5, either when the native gene was mutated or when an ectopic mutant copy was overexpressed (2). Again, DNA-sequencing confirmed the desired edit (Figure 3C), which was associated with drug resistance (Figure 3D), and the ability to introduce further synonymous edits (Figure 3C, Supplementary Figure S2D). From these experiments, we were able to estimate an allele replacement frequency of ∼10^–5^ for *Leishmania*. Indeed, all transfections, using either standard ssODNs or S-oligos, yielded drug-resistant populations for both *Leishmania* and *T. cruzi*. Using the assays described above, we found that editing and drug resistance were always ssODN-dependent, edits were always heterozygous, and drug resistance was always similarly increased in two or more independent edited clones. These results revealed the versatility and utility of oligo targeting in the trypanosomatid parasites associated with major neglected tropical diseases.

### Oligo targeting for site saturation mutagenesis

**Figure 4.**
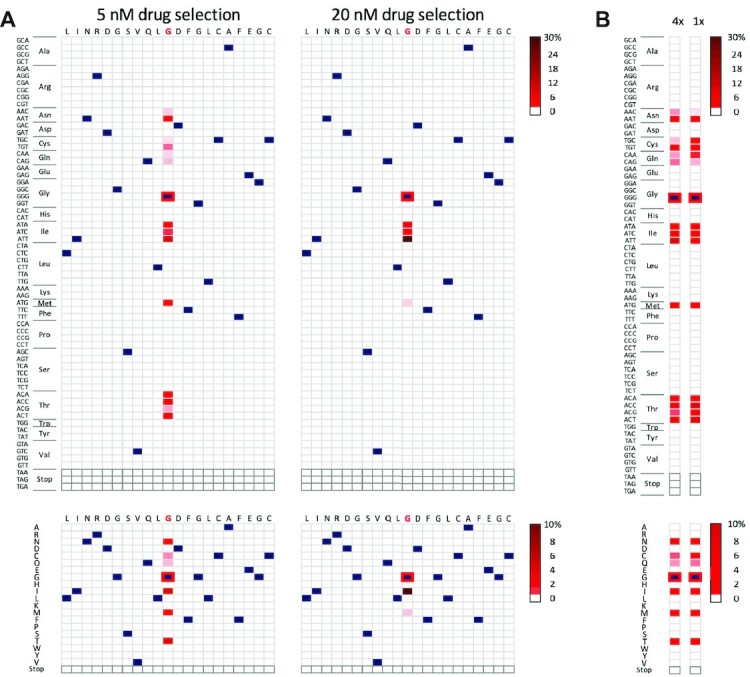
Oligo targeting for site saturation mutagenesis. (**A**) Amplicon sequencing and codon variant profiling following *Tb*CRK12 editing in *T. brucei* using a 53-b S-oligo with a centrally located degenerate G^492^ codon. The heatmaps show average proportions of edited codons within the targeted region following compound 2 selection, relative to mock-transfected control cells (no ssODN). The lower panels show averages for edited codons representing each amino acid. Dark blue indicates the wild type codons or amino acids while the unedited GGG/G^492^ allele is indicated by red outlining. Drug at either 5 nM or 20 nM was applied to two independent populations. (**B**) Data from panel A (5 nM drug selection), derived by averaging two pools of cells from four electroporations (4×), are compared to data derived in parallel but using a single population derived from a single electroporation (1×). Other details as in A except only the targeted codon/amino acid position is shown in this case.

Our maximal editing efficiency observed using oligo targeting, following MSH2 knockdown, remains 50-fold lower than the efficiency obtained previously using Cas9-based editing (10). Consequently, edited clones generated by oligo targeting are not readily isolated from populations in the absence of selection. The approach could be used to profile the variety of mutant amino acids and codons at a specific site that impact drug resistance, however. For such a site saturation mutagenesis approach, wild type *T. brucei* were mixed with an S-oligo containing a fully (64-fold) degenerate CRK12 G^492^ codon; G^492^ is adjacent to a conserved motif at the ATP binding site and mutation of this residue is a common route to kinase inhibitor resistance, including compound 2 resistance in *T. brucei* (3). Electroporated populations were grown in two different drug concentrations for five days, after which we used high-throughput amplicon sequencing to quantify those CRK12 edits that allowed the cells to tolerate drug pressure. The analysis revealed fourteen distinct resistance-conferring G^492^ codon edits encoding six distinct amino acids (Figure 4A). Following 5 nM drug selection, G^492^N, G^492^C, G^492^Q, G^492^I, G^492^M and G^492^T edits were observed (all possible codons in every case). Following 20 nM drug selection, the abundance of all edits encoding amino acids with polar neutral sidechains was diminished by >500-fold, while edits encoding amino acids with hydrophobic sidechains, G^492^I and G^492^M, persisted. Notably, editing was restricted to the target codon and all three nucleotides were edited in many of the new codons (Figure 4A). Thus, oligo targeting can be used to sample all possible codons at a given position, revealing edits and novel mutants with distinct drug resistance profiles, even following only a single electroporation (Figure 4B). Finally, we calculated how many clones likely represent each edited codon in these populations. A yield of 69 ± 32 resistant clones per electroporation, and fourteen among a possible sixty-four distinct codons observed following 5 nM drug selection, suggested an average of five clones representing each edited codon, or 20 when four electroporated populations were pooled.

Our findings demonstrate the utility of oligo targeting to generate focussed genetic diversity within practical timescales in the parasitic trypanosomatids (see Table 1). The approach can be used to confirm on-target activity and can facilitate the assessment of mutations that impact drug efficacy. This relatively simple, rapid, precise, versatile, scalable and low-cost mutagenesis approach can be used to further develop our understanding of drug resistance-associated mutations and structure-activity relationships, which should facilitate the rational optimization of drug efficacy and durability.

**Table 1.** Edited targets

Parasite	Target name	GeneID	Edit (aa, nt)	WT EC_50_ (RΔ)	Drug
*T. brucei*	CRK12	Tb927.11.12310	L^482^F, CTC-TTC	0.96 nM (33)	cpd 2 (3)
*T. brucei*	CRK12	Tb927.11.12310	G^492^x, GGG-NNN	0.96 nM (ND)	cpd 2 (3)
*T. brucei*	CPSF3	Tb927.4.1340	N^232^H, AAT-CAT	310 nM (4.8)*	acoziborole (4)
*T. cruzi*	proteasome β5	TCSYLVIO_004939	G^208^S, GGC-AGC	63.9 nM (666)	cpd 7 (6)
*L. donovani*	CRK12	LdBPK_090270	G^572^D, GGC-GAC	2.2 nM (3.3)	cpd 5 (2)

EC_50_, Half maximal effective concentrations; RΔ, fold-increase in EC_50_ in edited and resistant cells; cpd, compound; * EC_50_ and RΔ determined previously (4).

## DATA AVAILABILITY

Fastq files for the amplicon-seq analysis in this study have been deposited in the Short Read Archive (SRA) at https://www.ncbi.nlm.nih.gov/sra/ under BioProject ID PRJNA749253. The scripts to align the fastq files, qc control files and the scripts to extract the codon counts are available at https://github.com/mtinti/DH_Oligo-targeting and deposited in Zenodo (https://doi.org/10.5281/zenodo.5139694).

## Supplementary Material

gkac319_Supplemental_File
