# Supplementary material for: Oligo targeting for profiling drug resistance mutations in the parasitic trypanosomatids
Source: Nucleic Acids Res. 2022 May 7;50(14):e79. doi: 10.1093/nar/gkac319 (PMC9371896; doi:10.1093/nar/gkac319)
Supplement: gkac319_Supplemental_File [file gkac319_supplemental_file.pdf]

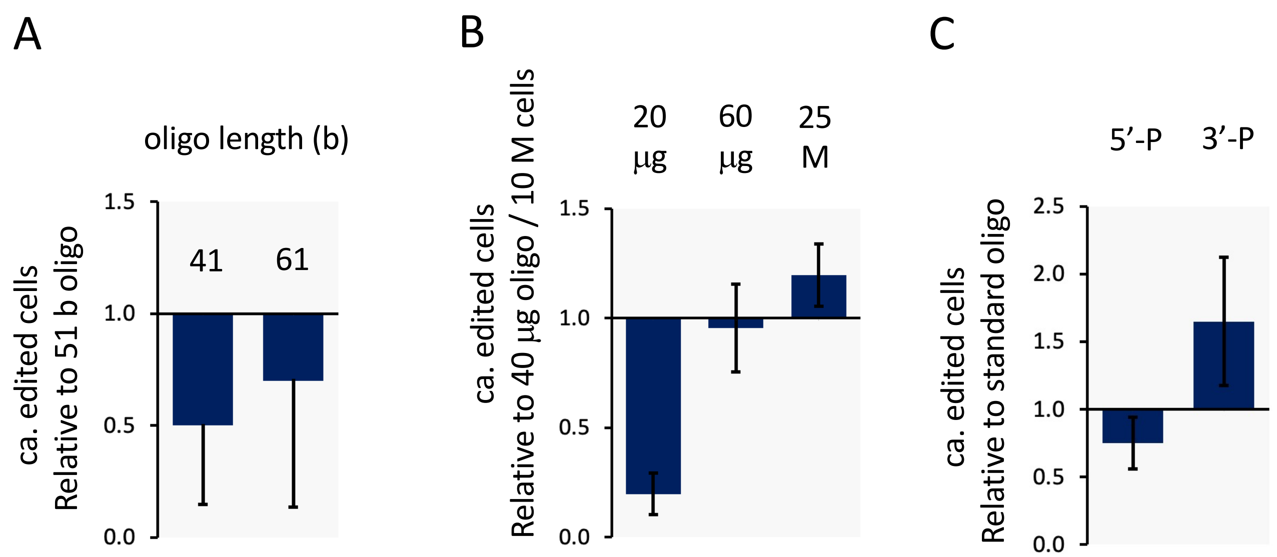

Figure S1. Oligo targeting in *T. brucei* - further assessment and optimization. *Tb*CRK12 $L^{482}F$, C-T editing. (**A**) The plot shows editing efficiency using reverse oligos of different length relative to a 51 b reverse oligo (see Figure 1B). (**B**) The plot shows editing efficiency using different quantities of 51 b reverse oligos or additional cells. (**C**) The plot shows editing efficiency using phosphorylated 51 b reverse oligos. Compound 2 selection was applied at 10 nM; n=2 for each assay; error bars, SD.

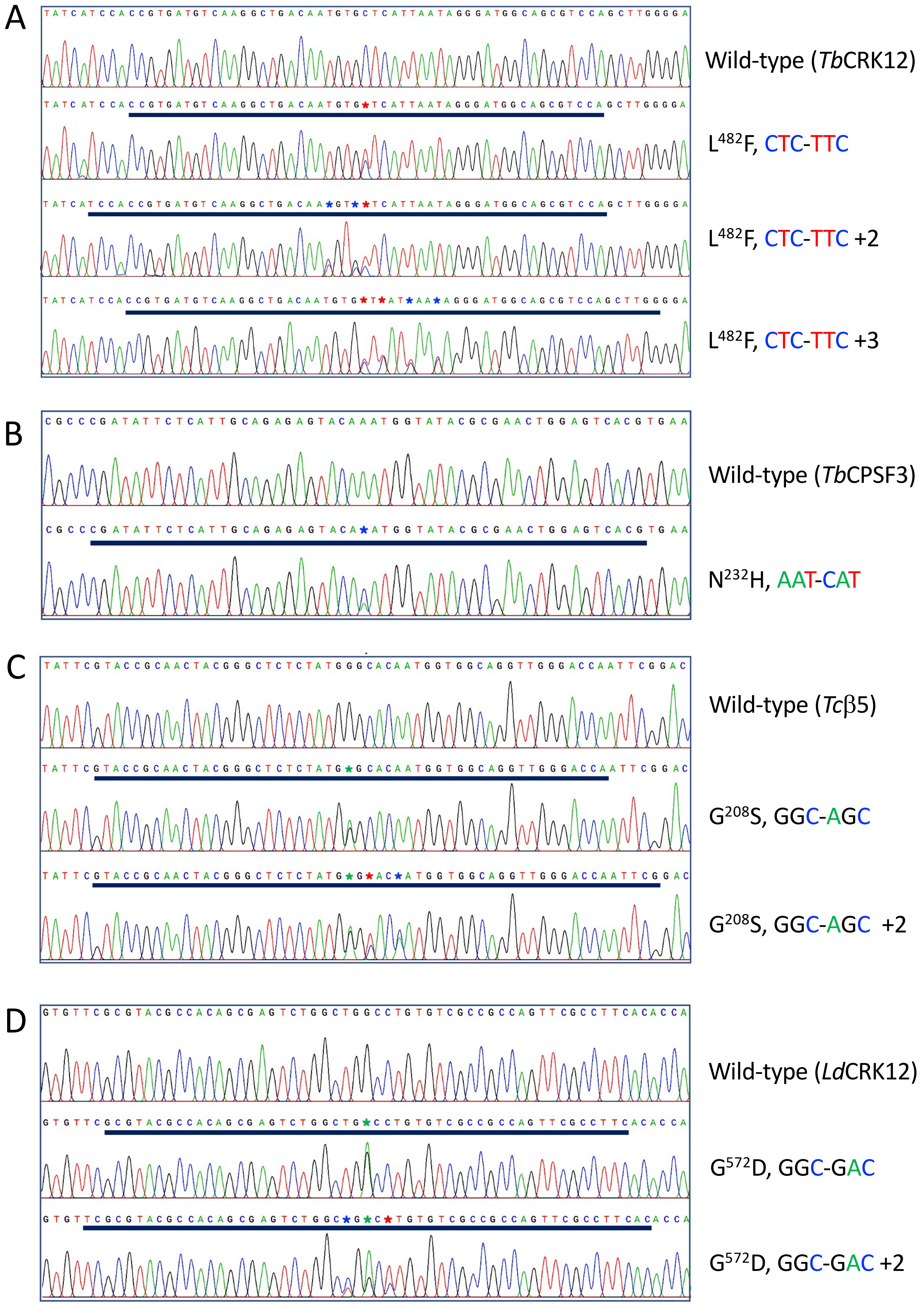

Figure S2. DNA sequencing confirmed specific editing. (**A**) *Tb*CRK12 $L^{482}F$. (**B**) *Tb*CPSF3 $N^{232}H$. (**C**) *Tc* proteasome β5 $G^{208}S$. (**D**) *Ld*CRK12 $G^{572}D$. The blue bars indicate the extent of each ssODN, all reverse direction. The number of additional synonymous changes are indicated on the right-hand side. A minimum of two independent clones were sequenced in each case and all sequences revealed the expected edits.

Gene name	Oligo name	Sequence
Tb CRK12	C12F21	TGACAATGTG**T**TCATTAATAG
Tb CRK12	C12R21	CTATTAATGA**A**CACATTGTCA
Tb CRK12	Tb-CRK12_L482F_51_F	CCGTGATGTCAAGGCTGACAATGTG**T**TCATTAATAGGGATGGCAGCGTCCA
Tb CRK12	Tb-CRK12_L482F_51_R	TGGACGCTGCCATCCCTATTAATGA**A**CACATTGTCAGCCTTGACATCACGG
Tb CRK12	C12F81	AATGGGTATCATCCACCGTGATGTCAAGGCTGACAATGTG**T**TCATTAATAGGGATGGCAGCGTCCAGCTTGGGGACTTCGG
Tb CRK12	C12R81	CCGAAGTCCCCAAGCTGGACGCTGCCATCCCTATTAATGA**A**CACATTGTCAGCCTTGACATCACGGTGGATGATACCCATT
Tb CRK12	C12R41	GCTGCCATCCCTATTAATGA**A**CACATTGTCAGCCTTGACAT
Tb CRK12	C12R61	CAAGCTGGACGCTGCCATCCCTATTAATGA**A**CACATTGTCAGCCTTGACATCACGGTGGAT
Tb CRK12	C12R51S	ZEEACGCTGCCATCCCTATTAATGA**A**CACATTGTCAGCCTTGACATCFOEG
Tb CRK12	C12R55*1	TGGACGCTGCCATCCCTATTAATGA**AG**AC**G**TTGTCAGCCTTGACATCACGGTGGA
Tb CRK12	C12R57*	CCAAGCTGGACGCTGCCATCCCT**G**TT**G**AT**A**A**A**CACATTGTCAGCCTTGACATCACGG
Tb CRK12	5P_C12R51	P-TGGACGCTGCCATCCCTATTAATGA**A**CACATTGTCAGCCTTGACATCACGG
Tb CRK12	C12R51_3P	TGGACGCTGCCATCCCTATTAATGA**A**CACATTGTCAGCCTTGACATCACGG-P
Tb CRK12	TbCRK12_51RLNA	TGGACGCTGCCATCCCTATTAATGA**+A**CACATTGTCAGCCTTGACATCACGG
Tb CRK12	C12Gly492_R53S	ZFFTAGGGATGGCAGCGTCCAGCTT**NNN**GACTTCGGTTTGTGCGCCTTTEFEG
Tb CRK12	CRK12-G492-AmpSeqF	TGCTGTGAAAAAGTTGCATGA
Tb CRK12	CRK12-G492-AmpSeqR	CAGGCGGGCGATAGTTAAG
Tb CPSF3	TbCPSF3-51R	CGTGACTCCAGTTCGCGTATACCAT**G**TGTACTCTCTGCAATGAGAATATCG
Tb MSH2	TbMSH2_RNAiF	GATCGGGCCCGGTACCATGGCGTACGGTTCGTATCC
Tb MSH2	TbMSH2_RNAiR	GATCTCTAGAGGATCCGCGACACAAACACCAATGCT
Tb MSH2	MSH2_qF	GTACCCCTAGCGGTCGATGT
Tb MSH2	MSH2_qR	AACGTGCGCAGATTTGAAAG
Tc proteasome β5	TcB5-51R	TGGTCCCAACCTGCCACCATTGTGC**T**CATAGAGAGCCCGTAGTTGCGGTAC
Tc proteasome β5	TcB5-51RS	ZEETCCCAACCTGCCACCATTGTGC**T**CATAGAGAGCCCGTAGTTGCGEZFC
Tc proteasome β5	TcB5-56R+2	CGAATTGGTCCCAACCTGCCACCAT**G**GT**A**C**T**CATAGAGAGCCCGTAGTTGCGGTAC
Tc proteasome β5	TcB5_PCRf	CGACAACTCTGGGGTTTCAT
Tc proteasome β5	TcB5_PCRr	TTGCATGAAAAATGGAACGA
Ld CRK12	LdC12-51R	GAAGGCGAACTGGCGGCGACACAGG**T**CAGCCAGACTCGCTGTGGCGTACGC
Ld CRK12	LdC12-51RS	EFFGGCGAACTGGCGGCGACACAGG**T**CAGCCAGACTCGCTGTGGCGTFOEC
Ld CRK12	LdC12-55R+2	GTGAAGGCGAACTGGCGGCGACACA**A**G**T**C**G**GCCAGACTCGCTGTGGCGTACGCGA
Ld CRK12	LdCRK12_Fw1	CCGCGCGATTACGGCTACA
Ld CRK12	LdCRK12_Rv3	CGTGTGGGGCGCCACTGA

Table S1. Oligonucleotides used in this study. All ssODNs used for editing are on a blue background. Non-synonymous edits are in bold red text and synonymous edits are in bold text.
